# A retrospective study of patients with blood culture-confirmed typhoid fever in Fiji during 2014–2015: epidemiology, clinical features, treatment and outcome

**DOI:** 10.1093/trstmh/trz075

**Published:** 2019-10-22

**Authors:** Aneley Getahun S, Christopher M Parry, John A Crump, Varanisese Rosa, Adam Jenney, Ravi Naidu, Kim Mulholland, Richard A Strugnell

**Affiliations:** 1 School of Public Health and Primary Care, College of Medicine, Nursing and Health Sciences, Fiji National University, Private mail bag, Suva, Fiji; 2 Doherty Institute for Infection and Immunity, University of Melbourne, 792 Elizabeth Street, Melbourne VIC 3000, Australia; 3 Institute of Infection and Global Health, University of Liverpool, The Ronald Ross Building, 8 West Derby Street, Liverpool, L69 7BE UK; 4 School of Tropical Medicine and Global Health, University of Nagasaki, 12, Nagasaki, 852-8102, Japan; 5 Centre for International Health, University of Otago, 55 Hanover Street, Dunedin 9016, New Zealand; 6 Fiji Centre for Communicable Diseases Control, Fiji Ministry of Health and Medical Services, Tamavua, Suva, Fiji; 7 School of Medical Science, College of Medicine, Nursing and Health Sciences, Fiji National University, Private mail bag, Suva, Fiji; 8 Colonial War Memorial Hospital, Fiji Ministry of Health and Medical Services, Waimanu Road, Suva, Fiji; 9 Murdoch Childrens Research Institute, Royal Children’s Hospital, Flemington Road, Parkville, Melbourne VIC 3052, Australia; 10 London School of Hygiene and Tropical Medicine, Keppel St, Bloomsbury, London WC1E 7HT UK

**Keywords:** antimicrobial susceptibility, clinical features, complications, Fiji, *Salmonella* Typhi, typhoid fever

## Abstract

**Background:**

Typhoid fever is endemic in Fiji. We sought to describe the epidemiology, clinical features and case fatality risk of blood culture-confirmed typhoid fever from January 2014 through December 2015.

**Methods:**

Blood culture-positive patients were identified from a typhoid surveillance line list. A standardised case investigation form was used to record data from patients’ medical records.

**Results:**

Of 542 patients, 518 (95.6%) were indigenous Fijians (iTaukei) and 285 (52.6%) were male. The median (IQR) age was 25 (16–38) y. Mean (SD) time from the onset of illness to admission was 11.1 (6.9) d. Of 365 patients with clinical information, 346 (96.9%) had fever, 239 (66.9%) diarrhoea, 113 (33.5%) vomiting, and 72 (30.2%) abdominal pain. There were 40 (11.0%) patients with complications, including 17 (4.7%) with shock, and 11 (3.0%) with hepatitis. Nine patients died for a case fatality risk of 1.7%. Of the 544 *Salmonella* Typhi isolates tested, none were resistant to first line antimicrobials; 3(0.8%) were resistant to ciprofloxacin and 5(1.4%) to nalidixic acid.

**Conclusions:**

In Fiji, most blood culture-confirmed typhoid fever cases were in young adults. Common clinical manifestations were fever and gastrointestinal symptoms. Further studies are required to elucidate the factors associated with complications and death.

## Introduction

Typhoid fever, an infection caused by *Salmonella enterica* subspecies *enterica* serovar Typhi (*Salmonella* Typhi), remains a common infection in low- and middle-income countries.^1^^,^^2^ In 2017, it was estimated to cause more than 10 million new cases worldwide,^3^ with an estimated 160 000 deaths.^4^

Fiji is an independent island nation in the South Pacific with a population in 2017 estimated at 884 887, of which 44.1% lived in rural areas.^5^ The two main ethnic groups are iTaukei, indigenous Fijians (56.8%) and Fijians of Indian descent (37.5%).^6^ Health services are provided mainly by the Ministry of Health and Medical Services (MoHMS). National health service delivery is through four medical divisions: central, western, northern, and eastern. Each division is further divided into subdivisions, medical areas and zones. There are three main public hospitals, one each in the central, northern and western divisions, and two specialist hospitals, both based in the central division. Primary healthcare is provided by 19 subdivisional hospitals, 86 health centres, and 97 nursing stations.^7^ Healthcare in public health facilities is provided free of charge. In addition, there is one private hospital in Suva in the central division. Several small privately owned medical centres and clinics exist in all divisions.

Passive laboratory surveillance for typhoid fever was established in Fiji 2004.^8^ Typhoid fever confirmed by culture of blood, stool, pus, or other sterile sites is reported within 24 h of confirmation by telephone to the Fiji Centre of Communicable Diseases Control (FCCDC) and to the treating medical officer in the respective health facility.^9^ In addition, all clinically diagnosed typhoid fever patients are reported through the national notifiable diseases surveillance system on a weekly basis.^10^ There has been an eightfold rise in laboratory-confirmed cases of typhoid fever detected by passive surveillance from 5.1/100 000 in 2004^8^ to 42.1/100 000 in 2011.^11^ Frequent outbreaks have also been reported since 2008.^12^^,^^13^ A review of surveillance reports from 2008–2012 found that most culture-confirmed typhoid fever was among young adults with a median age of 24 y and that 95% were indigenous Fijians known as iTaukei.^12^ Crude typhoid fever incidence was highest among people aged 15–29 y at 64/100 000/y.^11^^,^^12^ In 2010, the Fiji MoHMS conducted Vi polysaccharide mass vaccination of 65 015 people after a typhoid fever outbreak that followed tropical cyclone Tomas.^11^^,^^14^ The same year, MoHMS revised the national typhoid fever treatment guidelines, with oral ciprofloxacin 500 mg twice daily for 5 d being recommended for uncomplicated typhoid fever and amoxicillin (75 to 100 mg/kg for 14 d) for pregnant women.^14^ There have been limited recent data regarding the clinical features, complications, and case fatality risk (CFR) of typhoid fever in Fiji. Studies from the 1980s reported high CFR ranging from 2.2 to 5.9%.^15–17^ The present study was conducted to provide up-to-date evidence on the clinical features and case fatality in typhoid fever patients as well as the antimicrobial susceptibility patterns of Fiji *Salmonella* Typhi isolates.

## Materials and methods

Study participants were identified from the FCCDC surveillance line list of patients with culture-confirmed *Salmonella* Typhi infection. The line list incorporates both outbreak and sporadic cases of culture-confirmed typhoid fever. We performed a retrospective review of medical records of patients with blood culture-confirmed typhoid fever from January 2014 through December 2015. A standardised data collection form was used to record demography- (age, gender, ethnicity, residential location), clinical- (symptoms, signs, duration of illness, complications, laboratory results), and treatment-related information (antimicrobials used, antimicrobial susceptibility patterns of isolates, duration of hospital stay and death from typhoid). Typhoid fever deaths were, identified from a review of medical records of cases reported during the passive surveillance. Additional information on deaths was obtained from the patient information system (PATIS plus), which is an electronic database that codes mortality according to the International Classification of Diseases, 10th revision (ICD 10) through an automated system called Iris (version 4.0).^18^ The ICD 10 code used for typhoid deaths was A01.0.

Any typhoid fever patient or typhoid-attributable death diagnosed on clinical suspicion only or laboratory-confirmed by only stool culture was excluded.

Consistent with the Fiji 2010 national typhoid management guidelines, an outbreak of typhoid fever was defined as a sudden increase in the number of typhoid fever cases, or the identification of two or more suspected or confirmed cases of typhoid fever in one month in a new area or village.^14^ The sudden increase implies any unusually high number of cases compared with the previous reporting period (e.g., the preceding week) or to the same week of previous years. The Fiji MoHMS staff use this threshold to notify or declare an outbreak. Data collection was performed from February 2017 through July 2018.

### Complications of typhoid

Complications of typhoid fever were defined by the presence of one or more of the following features: (1) gastrointestinal bleeding (the presence of occult blood, melena, or visible blood in the stool); (2) intestinal perforation (confirmed at surgery); (3) encephalopathy (delirium, obtundation or coma); (4) haemodynamic shock (systolic blood pressure <90 mm Hg and/or diastolic blood pressure <60 mm Hg, associated with tissue hypoperfusion); (5) myocarditis (tachycardia or bradycardia with an associated abnormality of the electrocardiogram or ultrasound evidence of a pericardial effusion); (6) hepatitis (as indicated by jaundice and/or hepatomegaly with serum transaminases two times above the normal range); (7) a clinical diagnosis of cholecystitis (right upper quadrant pain and tenderness without evidence of hepatitis).^19^^,^^20^

**Table 1:** Demographic characteristics of typhoid fever patients in Fiji, 2014–2015

Demography	n/total (%)	Mean crude incidence (per 100 000 population)
Gender		
Male	285/542 (52.6)	31.9
Female	257/542 (47.4)	29.3
Ethnicity		
iTaukei	518/542 (95.6)	-
Fijian of Indian descent	14/542 (2.6)	-
Other	10/542 (1.8)	-
Age group, y		
0–4	34/531 (6.4)	19.3
5–14	84/531(15.8)	25.6
15–24	147/531(27.7)	49.1
25–39	139/531(26.2)	35.8
40–59	108/531(20.3)	28.2
60+	19/531 (3.6)	12.0
Medical division		
Central	158/542 (26.2)	20.8
Eastern	6/542 (1.1)	7.6
Northern	198/542 (36.5)	74.2
Western	180/542 (33.2)	23.8
Level of management		
Divisional hospital	206/542 (38.0)	-
Subdivisional hospital	284/542 (52.4))	-
Health centre	56/542 (10.3)	-

### Laboratory methods

Blood and stool collected from health centres, subdivisional and divisional hospitals were cultured at divisional hospital microbiology laboratories and potential *Salmonella* spp*.* were identified using standard microbiological methods. Blood cultures were performed using the BaCT/ALERT 3D (Biomerieux, Marcy L’Etoile, France) system. Antimicrobial susceptibility testing to ampicillin, chloramphenicol, ceftriaxone, ciprofloxacin, nalidixic acid and trimethoprim-sulfamethoxazole was performed by disk diffusion and E-test (BioMerieux) according to the standards and interpretative criteria of the Clinical and Laboratory Standards Institute.^21^ The antimicrobial susceptibility results from the study period were compared with the results collected from the same laboratories in 2004 and 2005. ^8^

### Statistical analyses

Data were analysed using Microsoft Excel (Microsoft Corp., Redmond, WA, USA) and SPSS version 24 (IBM, Armonk, NY, USA). Overall and specific crude incidence of typhoid fever were calculated using population projections provided by the Fiji Bureau of Statistics (FBoS) for 2014 and 2015. Since FBoS data are not disaggregated for medical division, crude incidence rates by medical divisions and subdivisions were calculated using the 2014 and 2015 population estimates from the MoHMS. CFR was calculated by dividing the number of deaths in blood culture-confirmed typhoid patients in 2014 and 2015 by the total number of blood culture-positive typhoid patients reported during the same period (n=542) multiplied by 100. Demographic profile, antimicrobial susceptibility pattern and outcome were assessed among all cases (n=542). Analysis of common clinical presentations, complications, treatment with antimicrobials and duration of hospital stay was conducted among patients treated in divisional and subdivisional hospitals (n=365). Categorical variables were presented as proportions and the statistical significance of differences was determined using the χ^2^ test or Fisher’s exact test with a 95% level of confidence. Continuous variables were described as proportions using mean or median with SD or IQR. Bivariate analyses were performed to assess the clinical and laboratory features between children (aged <15 y) and adults (aged **≥**15 y) with statistical significance determined at 0.05%.

## Results

During the study period, 551 instances of culture-confirmed typhoid fever (*Salmonella* Typhi infection) were reported to the FCCDC. Of these, 542 (98.4%) were confirmed by blood culture and were included in the analysis. The demographic characteristics of blood culture-confirmed typhoid fever patients are shown in Table 1. Of the 542 patients, 285 (52.6%) were male and 518 (95.6%) were from the iTaukei ethnic group. The median (IQR) age was 25 (16–38) y. Children aged <15 y accounted for 118 (21.8%) of the patients. The crude incidence of typhoid fever from passive surveillance was 32.1 and 30.4/100 000 of the population in 2014 and 2015, respectively. The mean age-specific crude incidence was 49.1/100 000 per year among people aged 15 to 24 y and 12/100 000 per year among those aged ≥60 y. The northern division reported 198 blood culture-confirmed typhoid patients for a mean annual crude incidence of 74.2/100 000 per year. The western and central divisions had a mean annual crude incidence of 23.8/100 000 (n=180) and 20.8/100 000 (n=158), respectively. At subdivisional level, in 2014 the highest mean annual crude incidence of 150.3/100 000 was reported in Ra in the western division and in 2015 it was 170.0/100 000 in Bua in the northern division. Further analysis of data by the location of patient residence indicated several community outbreaks and clusters. Of 24 typhoid cases reported in the Bua subdivision in 2014, 18 (75%) were from outbreaks in five villages. Similarly, of 51 cases in the Suva and 15 cases in the Namosi subdivisions, 22 (43%) and eight (53%) were outbreak-associated, respectively. In 2015, in the northern and central divisions, patients reported from community outbreaks accounted for more than half of all cases, such as 11 (55%) in the Namosi, five (62.5%) in the Rewa and eight (72%) in the Naitasiri subdivisions.

**Table 2:** Clinical and laboratory features of typhoid fever patients in Fiji, 2014–2015

Features	Total n/total (%)	Aged <15 y n/total (%)	Aged ≥15 y n/total (%)	p-value
Signs and symptoms				
History of fever	349/360 (96.9)	82/84 (97.6)	267/276 (96.9)	NS
Diarrhoea	239/357 (66.9)	52/83 (62.7)	187/274 (68.2)	NS
Loss of appetite	185/356 (52.0)	48/81 (59.3)	137/275 (49.8)	NS
Rigors	182/353 (51.6)	22/81 (27.2)	160/272 (58.8)	<0.001
Headaches	154/353 (43.6)	18/80 (22.5)	136/273 (49.8)	<0.001
Vomiting	119/355 (33.5)	25/81 (30.9)	94/274 (34.3)	NS
Conjunctival pallor	80/317 (25.2)	19/73 (26.0)	61/244 (25.0)	NS
Abdominal pain	72/354 (20.3)	20/80 (25.0)	52/274 (19.0)	NS
Cough	64/358 (17.9)	20/83(24.1)	44/275(17.9)	NS
Jaundice	31/318 (9.7)	2/72 (2.8)	29/246 (11.0)	0.02^¶^
Constipation	17/360 (4.7)	6/84 (7.1)	11/276 (4.0)	NS
Organomegaly^†^	8/343 (2.3)	4/82 (4.9)	4/261 (1.5)	NS ^¶^
Haematology				
Thrombocytopenia^‡^	146/325 (44.9)	39/75 (52.0)	107/250 (42.8)	NS
Anaemia^*^	135/329 (41.0)	38/77 (49.4)	97/252 (38.5)	NS
Leukopenia^¥^	132/332 (39.8)	36/77 (46.8)	96/255 (37.6)	NS

NS: not significant

^¶^Fisher’s exact test; ^†^include hepatomegaly and splenomegaly; ^‡^platelet count <100 000 cells/l; ^*^haemoglobin <11 g/dl for those aged <15 y and haemoglobin <12 gm/dl for individuals aged ≥15 y; ^¥^white blood cell count <5000 x 10 cells/l^6^

**Table 3:** Comparison of antimicrobial resistance pattern among *Salmonella* Typhi isolates, Fiji, 2004–2005 and 2014–2015

Antimicrobial	Resistance (2004–2005)^7^ n/total (%)	Resistance (2014–2015) n/total (%)
Ampicillin	3/272 (1.1)	0/544 (0)
Chloramphenicol	2/272 (0.7)	0/544 (0)
Trimethoprim-sulfamethoxazole	2/263 (0.8)	0/544 (0)
Doxycycline	3/209 (1.4)	0/2 (0)
Nalidixic acid	0/207 (0)	5/361 (1.4)
Ciprofloxacin	Not done	3/393 (0.8)

Of 542 patients, 486 (89.7%) were treated in hospitals and 56 (10.3%) were treated in health centres. Clinical information was available for 365 (75.1%) patients who received treatment at divisional and subdivisional hospitals. The mean (SD) time from the onset of illness to admission was 11.1 (6.9) d. The time from onset to admission was 11.1 (SD 5.9) d for males and 10.5 (SD 5.8) d for females (p=0.384), 10.8 (SD 5.8) d for iTaukei and 12.6 (SD 7.0) for Fijians of Indian descent (p=0.376), and 8.4 (SD 5.9) d for those aged <15 y and 11.5 (SD 5.1) d for those aged ≥15 y (p<0.001). The clinical features of typhoid fever are summarised in Table 2. Fever was reported in 349 (96.9%) of patients. History of diarrhoea and loss of appetite were reported in 239 (66.9%) and 185 (52.0%) of patients, respectively. Among adult patients, 160 (58.8%) and 136 (49.8%) gave a history of rigors and headaches, respectively. On physical examination, conjunctival pallor was reported in 80 (25.2%) patients and jaundice was found in 31 (9.7%) of patients. Abnormal neurologic findings such as confusion, lethargy or delirium were reported in 21 (6.1%) of patients. At admission, 135 (41.0%) patients had anaemia (haemoglobin <12 g/dl in adults and <11 g/dl in children aged <15 y), of whom 10 had severe anaemia (haemoglobin <7 g/dl) or needed transfusion (n=8). Leukopenia (white blood cell count <5000 x 10 cells/l)^6^ and thrombocytopenia (platelet count <100 000 cells/l) were reported in 132 (39.8%) and 146 (44.9%) of patients at admission, respectively (Table 2).

Of 365 patients, 40 (11.0%) developed complications. Seventeen (4.7%) had hypovolemic shock, 11 (3.0%) hepatitis, nine (2.5%) gastrointestinal bleeding, four (1.1%) encephalopathy and one (0.3%) myocarditis. Intestinal perforation and cholecystitis were not reported. The mean time to admission among patients with complications was 12.7 (SD 6.9) d and 10.7 (SD 6.9) d in patients with no complications (p=0.137). All patients with complications were from the iTaukei ethnic group. The occurrence of complication did not differ by gender (11.9% in females and 10.1% in males, p=0.618) or age (8.2% in those aged <15 y and 11.8% in those aged ≥15 y, p=0.432). There was a total of nine deaths among blood culture-confirmed typhoid fever patients. The overall CFR was 1.7%. Among 413 adults, eight died for an adult CFR of 1.9%. Among 118 children, one died for a child CFR of 0.8%.

Of 365 typhoid fever patients, 290 (79.5%) were treated with ciprofloxacin for the mean duration of 6 (SD 2) d and 80 (21.9%) received ceftriaxone. Other drugs used for treatment of typhoid fever included parenteral ampicillin, oral amoxicillin and chloramphenicol. Concerning antimicrobial susceptibility, all *Salmonella* Typhi strains were susceptible to ampicillin, trimethoprim-sulfamethoxazole and chloramphenicol (Table 3). Resistance to nalidixic acid was identified in five (1.4%) of 361 isolates tested and to ciprofloxacin in three (0.8%) of 393 isolates tested. No multidrug-resistant (MDR) *Salmonella* Typhi was identified during the study period.

## Discussion

In a 2-y retrospective study we found that typhoid fever in Fiji was most common among adolescents and young adults and the iTaukei ethnic group. While typhoid fever disproportionately affects infants and children in high incidence settings,^2^^,^^22^ less than one quarter of typhoid fever cases occurred in those aged <15 y in our study. It is possible that the passive surveillance system for typhoid in Fiji under-ascertains typhoid fever in younger age groups through a reluctance to draw blood for culture in this group, low blood volume, and prior antimicrobial use.^23^

In our study, the average duration of illness at the time of seeking healthcare was 11 d, similar to a 1982 study from Fiji that reported a mean duration of illness prior to admission of 13 d.^16^ Other studies from Oceania reported comparable durations of illness prior to presentation.^24^^,^^25^ The clinical features of typhoid in our study were similar to those observed in other studies conducted in endemic countries in Asia, Africa, and the Pacific.^26–28^ Fever was the most frequently reported symptom among both children and adults. Consistent with other studies, rigors and headaches were commonly reported among adults.^28^ Besides the non-specific generalised symptoms, typhoid fever patients in Fiji had a range of gastrointestinal symptoms. Approximately two-thirds of patients gave a history of diarrhoea, half complained of anorexia, one in three reported vomiting and 20% had abdominal pain. Unlike other studies in Asia and Africa^28^ that showed a higher occurrence of diarrhoea among children, we did not demonstrate differences in the occurrence of diarrhoea between children and adults. However, constipation was uncommon. The frequent presentation with diarrhoea in Fiji may result in misdiagnosis as diarrhoeal disease. Physical examination findings were non-specific, with high temperature, and tachycardia reported in more than half of patients.

In our study, most patients had anaemia and thrombocytopenia at admission. The reported high prevalence of anaemia in children is consistent with results of the recent systematic review of typhoid fever clinical features.^28^ However, the prevalence of anaemia in adults was much higher than studies from other endemic settings.^28^ Anaemia is common in Fiji and the national nutrition survey conducted in 2015 showed an anaemia prevalence of 63.1% among children aged <5 y and 40.1% among adults.^29^ Our participants may have background anaemia from an underlying micronutrient deficiency.

Thrombocytopenia was not reported as a common presentation of typhoid fever. Its prevalence varied substantially between studies. Some studies in Asia reported a thrombocytopenia prevalence of 4.6–15%.^27^^,^^30^ A systematic review by Azmatullah et al.^28^ reported a higher prevalence of thrombocytopenia (platelet count <150 000) in sub-Saharan Africa (35%) and East Asia and the Pacific (27%). Typhoid fever patients in Fiji have similar symptoms and haematologic abnormalities such as anaemia and thrombocytopenia to patients suffering from other common febrile illnesses such as dengue and leptospirosis. This could pose a further challenge for case management in health centres and subdivisional hospitals as confirmatory tests are often available only in divisional hospitals or the public health laboratoryin Suva.

The proportion of patients with complications in our study was similar to the global estimate of 10–15%^1^; however, the pattern of complications differed. Shock, hepatitis, and anaemia predominated in our study. Intestinal perforation is a late complication that might occur after blood culture is no longer positive and therefore could have been missed in our cohort. The CFR of typhoid fever is widely estimated to be <1% with appropriate antimicrobial treatment.^1^ Country-level studies and systematic reviews reported a substantial variation in CFR by age group and geographic region.^28^ We found an overall CFR of 1.7% and CFR among adults of 1.9%, which is higher than the reported mortality in Asia. Further prospective studies are required to better understand the independent risk factors for typhoid fever mortality in Fiji.

Globally, the emergence and rapid spread of the often drug-resistant *Salmonella* Typhi H58 lineage has been associated with increased treatment failure and mortality.^27^^,^^31^ There is limited literature available on the antimicrobial susceptibility pattern of *Salmonella* Typhi strains in Fiji. In 2005, Dunn et al.^8^ reported a low prevalence of resistance to the first-line drugs ampicillin, chloramphenicol, and trimethoprim-sulfamethoxazole, ranging from 0.8 to 1.1% (Table 3); 10 y later, the prevalence of resistance to first-line drugs remained similar, but resistance to nalidixic acid had increased from 0 to 1.4%. In 2010, ciprofloxacin became the first-line drug for the treatment of typhoid fever in Fiji. Ciprofloxacin is a restricted drug that is not available over-the-counter from private or public pharmacies. The first ciprofloxacin-resistant strains were reported in 2014 (FCCDC surveillance, unpublished data). The rise in nalidixic acid resistance compared with 2004 is of concern as it is associated with decreased susceptibility to fluoroquinolones.^1^ Moreover, there might be under-reporting as approximately 30% of samples (mainly from the northern division) were not tested for nalidixic acid or ciprofloxacin susceptibility. Other studies in the Pacific also demonstrated a low prevalence of antimicrobial resistance.^24^^,^^25^ This could suggest that fluoroquinolone-resistant and MDR isolates of *Salmonella* Typhi have not yet emerged or been introduced to Fiji or other typhoid endemic islands in the Pacific.

Our study has several limitations. Being retrospective, we relied on obtaining data from patients’ medical records. These were not available for some patients. Furthermore, data were incomplete and were of variable quality for some patients for whom records were available. Complications such as intestinal perforation may have been missed as it can occur at a later stage when blood culture is negative. Crude incidence in our study was estimated using the data from passive surveillance, probably underestimating the scale of the typhoid fever problem. We were unable to report on prehospital antimicrobial use as it was not routinely documented. In addition, some selection bias may be present in the study due to the exclusion of patients whose medical folders were not available from the health facilities. As a result, we were unable to identify independent factors associated with fatality.

## Conclusions

Our study provides updated information on the clinical features of typhoid fever in Fiji. The majority of blood culture-confirmed typhoid fever cases were among young adults. Common clinical manifestations were fever and gastrointestinal symptoms, with a high prevalence of anaemia, thrombocytopenia, and complications such as shock and hepatitis among typhoid fever patients admitted to hospital. The reported prevalence of complications is high despite a low level of antimicrobial resistance; further studies are warranted to investigate the factors associated with complications. Our findings revealed that, using the Fiji MoHMS definition, the majority of typhoid fever cases were associated with outbreaks. As per the national typhoid management guidelines, proper investigation of such outbreaks is warranted to identify and treat subclinical cases, assess the role of unsafe water and unimproved sanitation facilities in transmission, and to search for chronic carriers of *Salmonella* Typhi who could be implicated in food or water contamination.^14^ We highlight the potential emerging resistance among *Salmonella* Typhi strains to nalidixic acid and fluoroquinolones. Sustained typhoid fever clinical and laboratory surveillance is vital to monitor this important disease threat, including the impact of prevention and control efforts.
